# Activation of salt-inducible kinase 2 promotes the viability of peritoneal mesothelial cells exposed to stress of peritoneal dialysis

**DOI:** 10.1038/cddis.2016.79

**Published:** 2016-07-21

**Authors:** H-H Wang, C-Y Lin, S-H Su, C-T Chuang, Y-L Chang, T-Y Lee, S-C Lee, C-J Chang

**Affiliations:** 1 Department of Pediatrics, Division of Pediatric Immunology and Nephrology, Taipei Veterans General Hospital, Taipei, Taiwan; 2 Department of Pediatrics, Faculty of Medicine, School of Medicine, National Yang-Ming University, Taipei, Taiwan; 3 Institute of Emergency and Critical Care Medicine, School of Medicine, National Yang-Ming University, Taipei, Taiwan; 4 Graduate Institute of Clinical Medical Science, College of Medicine, China Medical University, Taichung, Taiwan; 5 Clinical Immunological Center and Division of Pediatric Nephrology, Children's Hospital of China Medical University, Taichung, Taiwan; 6 Institute of Molecular Medicine, National Taiwan University, Taipei, Taiwan; 7 Department of Integrated Diagnostics and Therapeutics, National Taiwan University Hospital, Taipei, Taiwan; 8 Institute of Biological Chemistry, Academia Sinica, Taipei, Taiwan; 9 Graduate Institute of Biochemical Sciences, College of Life Science, National Taiwan University, Taipei, Taiwan

## Abstract

Maintaining mesothelial cell viability is critical to long-term successful peritoneal dialysis (PD) treatment. To clarify the viability mechanism of peritoneal mesothelial cells under PD solutions exposure, we examined the mechanisms of cellular response to this stress conditions. Here we report that the proteasome activity is inhibited when treated with PD solutions. Proteasome inhibition-mediated activation of salt-inducible kinase 2 (SIK2), an endoplasmic reticulum-resident protein, is important for mesothelial cell viability. SIK2 is mobilized to promote autophagy and protect the cells from apoptosis under PD solution or MG132 treatment. Immunofluorescence staining showed that SIK2 is colocalized with LC3B in the autophagosomes of mesothelial cells treated with PD solution or derived from patients undergoing PD treatment. SIK2 activation is likely via a two-step mechanism, upstream kinases relieving the autoinhibitory conformation of SIK2 molecule followed by autophosphorylation of Thr175 and activation of kinase activity. These results suggest that activation of SIK2 is required for the cell viability when proteasome activity is inhibited by PD solutions. Maintaining or boosting the activity of SIK2 may promote peritoneal mesothelial cell viability and evolve as a potential therapeutic target for maintaining or restoring peritoneal membrane integrity in PD therapy.

Peritoneal dialysis (PD) is an established treatment for end-stage renal disease.^1^ Successful treatment depends on the preserved functional integrity of the peritoneal membrane. Peritoneal mesothelial cells line the surface of the peritoneal membrane and form the permeability barrier across which ultrafiltration and diffusion occurs. Peritoneal mesothelial cells also have important roles in mediating leukocyte trafficking, maintenance of peritoneal homeostasis, antigen presentation, inflammation and tissue repair.^2, 3, 4, 5, 6^ Approximately 20–30% of patients treated with PD gradually lose peritoneal membrane function, which compromises the efficiency of dialysis and leads to treatment failure.^7^ Partial or total disappearance of mesothelial cells, loss of peritoneal membrane integrity and peritoneal fibrosis develop in a majority of these patients.^8, 9, 10^ Thus, prolonging and maintaining mesothelial cell survival is critical for long-term preservation of the peritoneum as a dialyzing organ.^11^

Peritoneal mesothelial cells are continuously exposed to stress condition of low pH, hyperosmotic and glucose-enriched PD solution during PD therapy.^12^ Biopsies of peritoneum from patients on PD showed ultrastructural alterations in the mesothelium of increasing development of rough endoplasmic reticulum (ER) and decreasing in surface microvilli.^13, 14^ These bioincompatible PD solutions provoke mesothelial cell injury, and mesothelial denudation is observed in PD patients.^8, 9^ While mesothelial cell death can be induced by bacterial peritonitis,^15^ the mesothelial cells remain viable in the bioincompatible PD solutions and can be cultured.^16^ The viable mesothelial cells in PD solutions may have the potential to re-establish the mesothelium, prolong the mesothelium function and lead to the success of PD treatment.^17^ Therefore, modulating mesothelial cell viability will make the long-term success of the PD technique possible.^18, 19^ However, how the mesothelial cells cope with the stress caused by continuous exposure to the bioincompatible PD solution remains unknown.

Cells respond to stress in various ways ranging from activation of pathways that promote survival to the initiation of cell death that eliminates damaged cells.^20^ The ubiquitin-proteasome system (UPS) and autophagy are two major systems of cellular catabolism. The proteasome is a multicatalytic enzyme complex that has a central role in degradation of damaged or misfolded proteins, and regulation of proteins that control cell-cycle progression and apoptosis.^21^ Inhibition of proteasome function disrupts the proteins degradation and results in cell-cycle arrest and apoptosis. Autophagy is a catabolic process in which cellular organelles and protein aggregates are delivered to the lysosomal compartment for degradation. Autophagy also has important functions in antigen presentation, elimination of microbes and regulation of development and cell death.^22^ Thus, autophagy and the UPS are critical to the maintenance of cellular homeostasis. For a long time, the above were regarded as two independent pathways because of the different machinery, specificities and elements of control. However, recent studies showed cross-talk between the UPS and autophagy systems.^22, 23, 24, 25^ Although the functional connection between the two systems is not well understood, impairment of proteasome activity was found to activate autophagy and salt-inducible kinase 2 (SIK2),^26^ p62, NBR1 (neighbor of BRCA1 gene 1), HDAC6 (histone deacetylase 6) and Alfy have been reported to be the linkers of the two.^27^ This suggests a coordinated and complementary relationship between the two degradation systems under cellular stress conditions. The UPS and autophagy regulate cell stress response and viability; however, their role in PD and mesothelial survival is still unknown.

SIK2 is a member of the AMP-activated protein kinase (AMPK) family. It has been shown that these kinases are important mediators of energy and stress responses. SIK2 has a regulatory role in gene expression in response to hormones and nutrients.^28, 29, 30, 31, 32^ SIK2 is also linked to melanogenesis,^33^ neuronal survival,^34^ corticotropin-releasing hormone transcription^35^ and mitotic progression in cancer cells.^36, 37^

SIK2 performs important regulatory functions in ERAD (ER-associated degradation) via interaction with VCP/p97 and in autophagy when proteasome activity is impaired.^38, 39^ Phosphorylation of SIK2 on Thr175 is the hallmark of its kinase activation. Although LKB1 is known to activate 13 kinases of the AMPK family in *in vitro* assays,^40^ the basal level of SIK2-Thr175 phosphorylation still persists in LKB1-null HeLa cells, thus implying additional regulatory mechanisms. Another potential key determinant of the post-translational regulation of SIK2 kinase lies in protein stability and protein–protein interaction. We have studied SIK2 activation by inhibition of proteasome and formation of complex with protein phosphatase 2 (PP2A).^39^ The SIK2–PP2A complex preserves both kinase and phosphatase activities. However, issues regarding other modes of modifications and regulation for this multifunctional kinase in cells (e.g., somatic diploid cells) other than cancer cell lines are currently unresolved.

In this report, we found a hitherto unrecognized requirement of SIK2 activity for promoting viability of mesothelial cells during PD. We have found that SIK2 is an ER lipid raft-resident protein. Proteasome inhibition in mesothelial cells caused by PD solution was observed. Under these conditions, SIK2 is mobilized to promote autophagy and protect the cells from apoptosis. Collectively, these results demonstrate novel cellular roles of SIK2 in ERAD and autophagy in clinically relevant conditions. Maintaining or boosting the activity of SIK2 may promote peritoneal mesothelial cells viability and protect mesothelial cells against stress from PD solution exposure during PD therapy.

## Results

### Subcellular localization of SIK2 in peritoneal mesothelial or foreskin fibroblast cells

**Figure 1:**
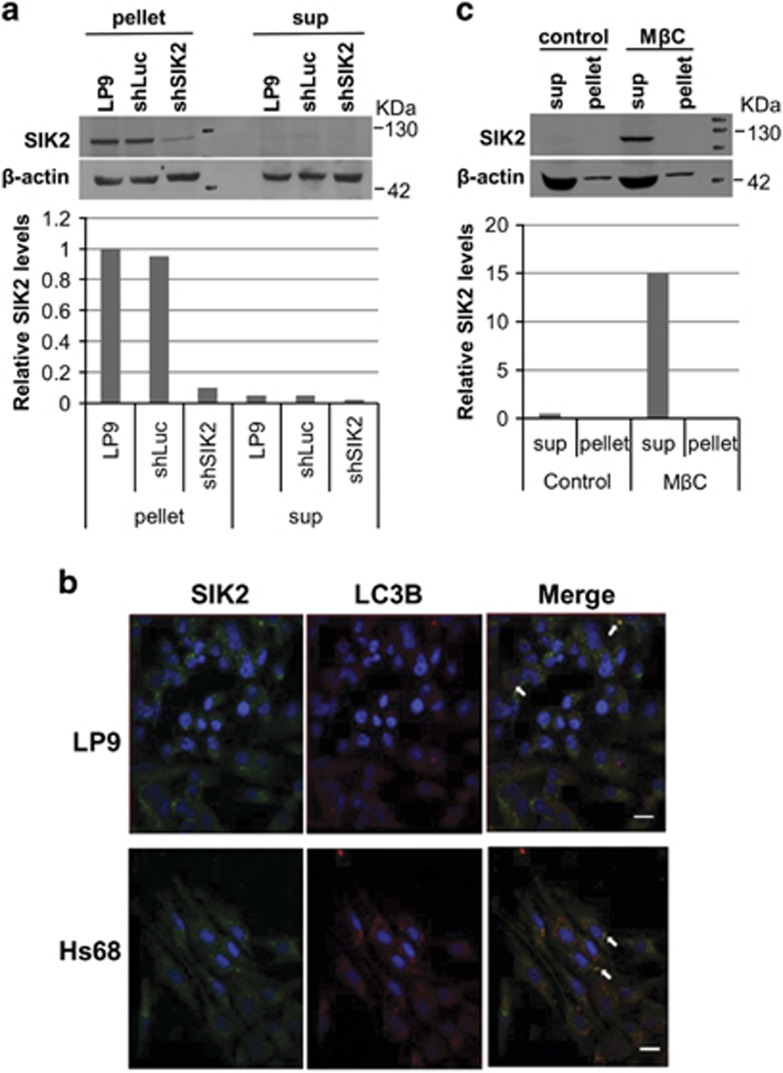
Subcellular localization of SIK2 in peritoneal mesothelial LP9 cells. (**a**) LP9 cells were infected with lentivirus-shLuc or -shSIK2 in a 6-cm Petri dish for 72 h. Cells were collected and extracted by WCE buffer. After centrifugation, both pellets and supernatants were subjected to western blot by probing with anti-SIK2 (moncolonal antibody (mAb) 15G10) and rabbit anti-*β*-actin antibodies. The relative protein levels of SIK2 were displayed by normalizing with the level of *β*-actin in the lower panel. (**b**) Immunofluorescence staining of SIK2 in LP9 (upper) and Hs68 (lower) cells. SIK2 and LC3B were colocalized but very faint without induction by stress. The arrows indicate the yellow spots of merged SIK2 and LC3B signals. Scale bars represent 20 *μ*m. (**c**) SIK2 was solubilized by M*β*C. Control or M*β*C-treated LP9 cells were extracted by WCE buffer and soluble and insoluble fractions were analyzed by western blotting with anti-SIK2 and anti-*β*-actin. The relative protein levels of SIK2 were quantitated by normalizing with the level of *β*-actin

SIK2 has been demonstrated to localize in the cytoplasm and peripheral membranes of ER in the interphase of cancer cell lines.^37, 39^ We further study the subcellular distribution of SIK2 in non-cancer cells (peritoneal mesothelial and foreskin fibroblast Hs68 cells) by biochemical fractionation and immunofluorescence staining. SIK2 is mainly located in the pellet of non-ionic detergent-containing buffer-extractable fraction from mesothelial and Hs68 cells (Figures 1a and b). Contrary to these cells, SIK2 from the cancer cell lines (e.g., 293T cells) is readily solubilized with that buffer.^39^ To further dissect the sub-ER location of SIK2, we treated mesothelial cells with methyl-*β*-cyclodextrin (M*β*C), a reagent used to selectively extract cholesterol from the membrane. Control and M*β*C-treated cells were separated into soluble and insoluble fractions. SIK2 indeed appeared mainly in the M*β*C soluble fraction (Figure 1c). These results suggest that SIK2 may exist in the cholesterol-rich membrane fraction (possibly lipid raft microdomain) of ER in mesothelial cells. Together, the existence of SIK2 in the non-ionic detergent-insoluble fraction and its specific decrease with knockdown of SIK2 by lentiviral-shSIK2 and its solubilization by M*β*C suggest that SIK2 is indeed mainly located in the membrane of peritoneal mesothelial cells.

### Peritoneal mesothelial cell SIK2 is released from ER by PD solution

**Figure 2:**
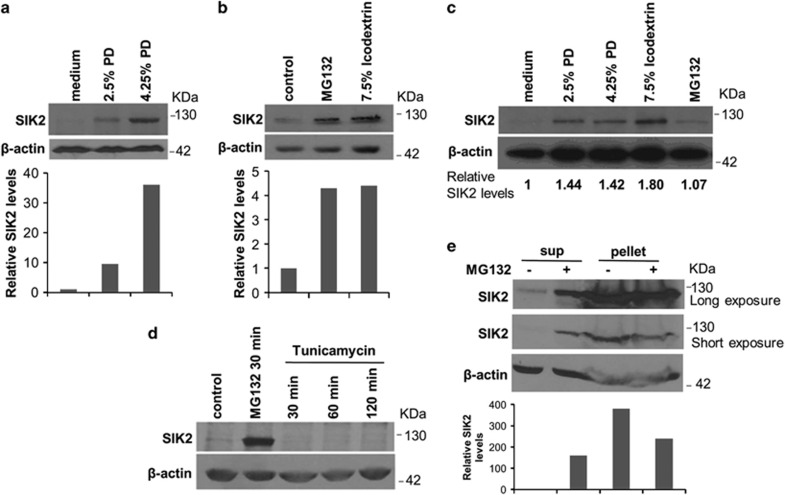
Regulation of SIK2 solubility. (**a**) PD solution treatment increases SIK2 solubility. LP9 cells were treated with 2.5% or 4.25% dextrose PD solution for 2 h. The supernatants from extraction with WCE buffer were analyzed by western blotting with anti-SIK2 and anti-*β*-actin antibodies. The quantitative result showed below. (**b**) MG132 or icodextrin treatment increases SIK2 solubility. Hs68 cells were treated with 25 *μ*M MG132 or 7.5% icodextrin PD solution for 2 h. WCE buffer-extracted supernatants were subjected to western blotting by anti-SIK2 and anti-*β*-actin. The quantitative SIK levels were shown below. (**c**) Human peritoneal mesothelial cells derived from dialysis effluents were treated with 2.5% dextrose, 4.25% dextrose, 7.5% icodextrin PD solution or 25 *μ*M MG132 for 2 h. The WCEs were isolated for western blotting analysis with anti-SIK2 and anti-*β*-actin. (**d**) ER stress-inducing agent tunicamycin treatment has no significant effect on SIK2 solubility. LP9 cells were treated with tunicamycin (2 *μ*g/ml in DMSO) for 30, 60 and 120 min or with MG132 (25 *μ*M) for 30 min. Cell lysates were analyzed by using anti-SIK2 and anti-*β*-actin antibodies. (**e**) LP9 cells were treated with 25 *μ*M MG132 for 30 min. The WCEs were prepared and centrifuged at 12 000 r.p.m. for 20 min at 4 °C. The supernatant (soluble) and pellet (insoluble) fractions were analyzed by western blotting

Stress response is expected when mesothelial cells are exposed to bioincompatible PD solution. Markers for the ER stress response such as increase of BiP, XBP-1s and activated ATF6 were probed. None of these markers was affected by PD solution in a significant manner (data not shown). We turned our attention to a newly defined ER-resident protein, SIK2. Our previous investigations indicated that SIK2 performs critical regulatory functions under stress conditions, such as ERAD and autophagy.^39^ To study the role of SIK2 in peritoneal mesothelial cells in stress condition, cell-free extracts were prepared from control or PD solution-treated mesothelial cells and probed with anti-SIK2 monoclonal antibody. The results showed that SIK2 is readily extracted from cells treated with 2.5% or 4.25% of dextrose PD solutions but not from control cells (Figure 2a). To examine whether 7.5% icodextrin PD solution and proteasome inhibitor MG132 have similar effect, we performed additional western blot experiments. Similar to 4.25% PD solution, both 7.5% icodextrin and MG132 could facilitate the non-ionic buffer extraction of SIK2 (Figure 2b). As shown in Figure 2c, human peritoneal mesothelial cells derived from dialysis effluents also responded to PD solution to increase SIK2 levels in non-ionic buffer extracts.

To further clarify whether ER stress-inducing agent such as tunicamycin could also result in increased SIK2 in the extractable fraction, we probed SIK2 in extracts from MG132- or tunicamycin-treated mesothelial cells. In sharp contrast to MG132, tunicamycin treatment had no or minimal effect on the extraction of SIK2 (Figure 2d). To address the relative distribution of SIK2 in the soluble and insoluble fractions, we probed SIK2 in these fractions prepared from control and MG132-treated mesothelial cells. MG132 treatment caused the release of significant amount of SIK2 in contrast to the exclusive presence of SIK2 in the insoluble fraction from the controls (Figure 2e).

### Inhibition of proteasome activity by PD solution

**Figure 3:**
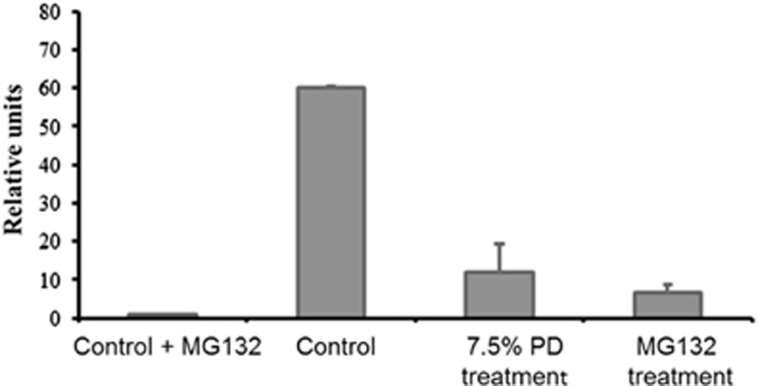
Inhibition of proteasome by PD solution. Hs68 cells were treated with dimethyl sulfoxide (DMSO) (vehicle control), 7.5% icodextrin PD solution or 25 *μ*M MG132 for 2 h. Cell lysates extracted by WCE buffer were subjected to proteasome activity assays. An aliquot of control cell lysate was incubated with 20 *μ*M MG132 just before incubating with the substrate (control+MG132)

Having observed that SIK2 becomes non-ionic detergent-extractable upon treatment with PD solution or MG132 but not by ER stressor, we reasoned that PD solution might inhibit proteasome similar to MG132. To test this possibility, we performed proteasome activity assay of control, 7.5% icodextrin PD solution and MG132-treated Hs68 cells. Treatment of these cells with either MG132 or 7.5% icodextrin PD solution for 2 h resulted in inhibition of proteasome activity (Figure 3). These results suggest that one of the immediate effects of PD solution is inhibition of proteasome activity.

### Autophagosome formation was triggered in peritoneal mesothelial cells separated from PD effluents of PD patients

**Figure 4:**
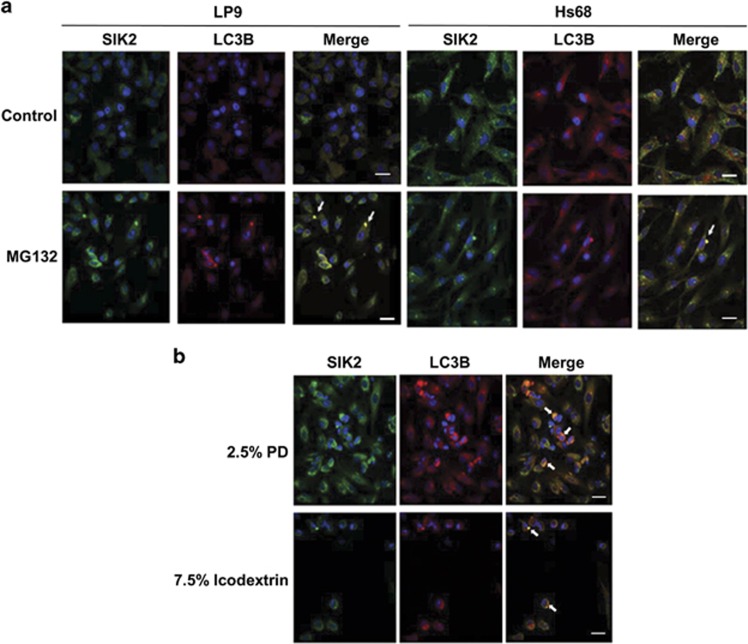
PD or MG132 induces autophagosome formation. (**a**) Immunofluorescence staining of LP9 cells (right) or Hs68 cells (left) under MG132 treatment. LP9 or Hs68 cells were cultured on a coverslip and treated with control solvent (dimethyl sulfoxide (DMSO)) or with 25 *μ*M MG132 for 2 h. Cells were detected by anti-SIK2 and anti-LC3B antibodies. (**b**) Immunofluorescence of mesothelial cells recovered from 2.5% dextrose (upper) or 7.5% icodextrin (lower) PD solutions from patient undergoing PD. The recovered mesothelial cells from PD solutions were cultured on a coverslip and detected with anti-SIK2 and anti-LC3B antibodies. Nuclei were visualized by Hoechst staining. The arrows indicate the yellow spots of merged SIK2 and LC3B signals. Scale bars represent 20 *μ*m

Proteasome and lysosome are the two major systems for coordinated protein catabolism during stress response. Our previous studies suggest that when proteasome activity is impaired, SIK2 facilitates autophagy.^26^ To address whether PD solution-mediated proteasome inhibition affects autophagy, we performed immunofluorescence staining on MG132-treated peritoneal mesothelial cell, Hs68 cells and peritoneal mesothelial cells separated from patients undergoing PD with SIK2 and microtubule-associated protein 1A/1B-light chain 3 (LC3) antibodies. MG132 treatment resulted in the colocalization of SIK2 and LC3B to the inclusion bodies (Figure 4a). We further examined the peritoneal mesothelial cells from patient treated with 2.5% dextrose or 7.5% of icodextrin PD solution by immunofluorescence staining of SIK2 and LC3B. Indeed, both PD solutions caused the elevation of SIK2 and the colocalization of SIK2 and LC3B in the autophagosomes (Figure 4b).

### Proteasome inhibition-mediated SIK2 activation is important for promoting cell viability

**Figure 5:**
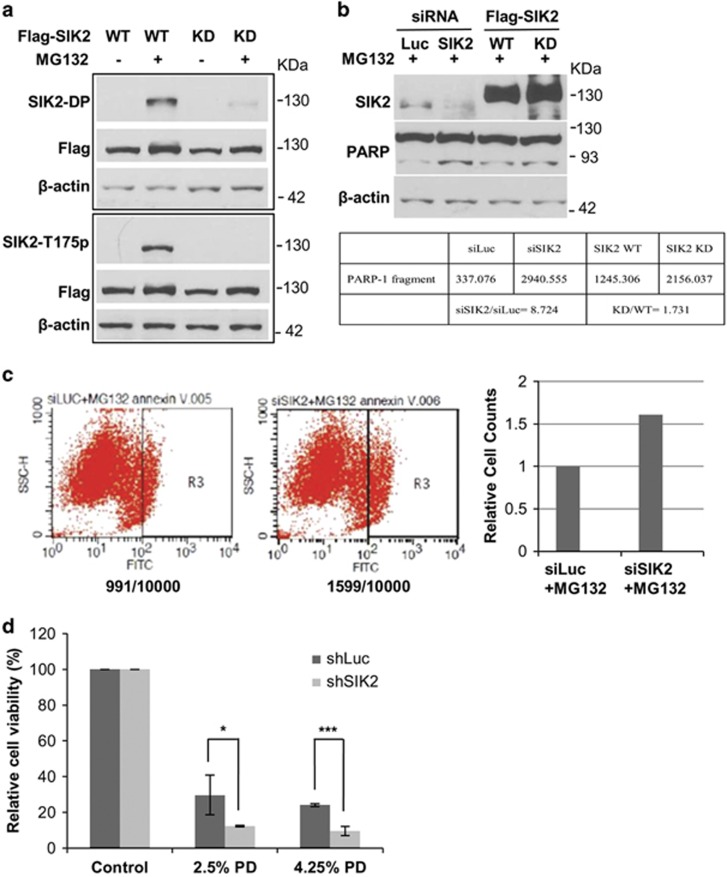
SIK2 is crucial for the cell survival upon MG132 treatment. (**a**) SIK2 is activated and stabilized by MG132. The 293T cells were transfected with Flag-SIK2-WT or Flag-SIK2-KD followed by treatment with or without 5 *μ*M MG132 for 16 h. Western blotting was performed with anti-phospho-SIK2-Ser545p/550p (SIK2-DP), anti-Flag, anti-phospho-SIK2-Thr175p (SIK2-T175) and anti-actin antibodies. (**b** and **c**) Knockdown of SIK2 increases cell apoptosis in response to MG132 treatment. (**b**) The 293T cells were transfected with short hairpin RNA (shRNA) against luciferase or SIK2, or overexpressed with Flag-SIK2-WT or Flag-SIK2-KD. After 24 h, cells were treated with vehicle solvent or 5 *μ*M MG132 for 16 h. The harvested cell lysates were detected with anti-SIK2, anti-PARP-1 and *β*-actin antibodies. The quantification results were shown below. (**c**) The 293T cells were transfected with shRNA against luciferase or SIK2. After 24 h, cells were treated with vehicle solvent or 5 *μ*M MG132 for 16 h. Cells were then isolated by binding with Annexin V-FITC and analyzed by flow cytometry. The Annexin V-positive cells were counted and the quantitative result is displayed in the right panel. (**d**) Control (shLuc) and SIK2 knockdown (shSIK2) LP9 cells were treated with 2.5% or 4.25% of dextrose PD solution for 2 h. Cells were harvested for trypan blue staining to monitor cell viability. This experiment was independently repeated three times

Our previous results demonstrated that SIK2 is important for regulating autophagy when proteasome is inhibited.^26^ To address whether the turnover of SIK2 *per se* depends on proteasome, we treated 293T with MG132. MG132 treatment specifically stabilizes the protein level of SIK2 and its localization to the inclusion bodies (Figures 5a and b). These results suggest that SIK2 turnover is mediated by proteasome. The stability of kinase-dead mutant of SIK2 (SIK2-KD, i.e., K49M, an ATP-binding deficient mutant) is strikingly different from that of wild-type SIK2 (SIK2-WT) with or without MG132 treatment. SIK2-KD is much more stable than SIK2-WT.^39^ Our previous results indicated that SIK2-KD is sequestered to the inclusion bodies and thus resistant to proteasome degradation.^39^ Together, these results not only indicate that the level of SIK2 is regulated by UPS but also show the functions of SIK2 in autophagy when UPS is compromised.

The hallmark of SIK2 activity is the phosphorylation of threonine-175 located in the activation loop. When cells were treated with MG132, the level of phosphorylated SIK2/Thr175 is highly elevated (Figure 5a). Accompanied to the elevation of SIK2/Thr175, SIK2/Ser545 and SIK2/Ser550 were also phosphorylated by MG132 treatment (Figure 5a). Strikingly, phosphorylation of SIK2/Thr175 of SIK2-KD cannot be induced by MG132 treatment, whereas the phosphorylation levels of SIK2/Ser545 and SIK2/Ser550 were greatly reduced but detectable as compared with that of SIK2-WT (~3.3:1) (Figure 5a). These results suggest that the access to SIK2-KD by MG132-induced kinase is minimal due largely to its location in the inclusion bodies. The lack of SIK2/Thr175 phosphorylation of SIK2-KD suggests that SIK2/Thr175 of SIK2-WT is autophosphorylated following the activation of upstream kinase(s) (e.g., LKB1) by MG132.

Having observed the parallelism of MG132-induced phosphorylations of SIK2/Thr175, SIK2/Ser545 and SIK2/Ser550, we performed experiments to identify the kinase involved in the phosphorylation of SIK2/Ser545 and SIK2/Ser550. Among the pharmacological agents that were tested, only SP600125 was effective in inhibiting these phosphorylations (data not shown). These results suggest that JNK may be the kinase responsible for the phosphorylation of SIK2/Ser545 and Ser550. Treatment with MG132 of SIK2 knockdown 293T cells resulted in enhanced apoptosis as demonstrated by the cleavage of PARP-1 (Figures 5b and c). Overexpression of SIK2-WT protects 293T cells from MG132-triggered apoptosis much more than SIK2-KD (Figure 5b). Consistent with these results, SIK2 knockdown LP9 cells also decreased cell viability under PD solution treatment (Figure 5d). These results suggest that the level/activity of SIK2 is important to the cell viability when UPS is inhibited.

### The kinase activity of SIK2 is negatively regulated by PP1

**Figure 6:**
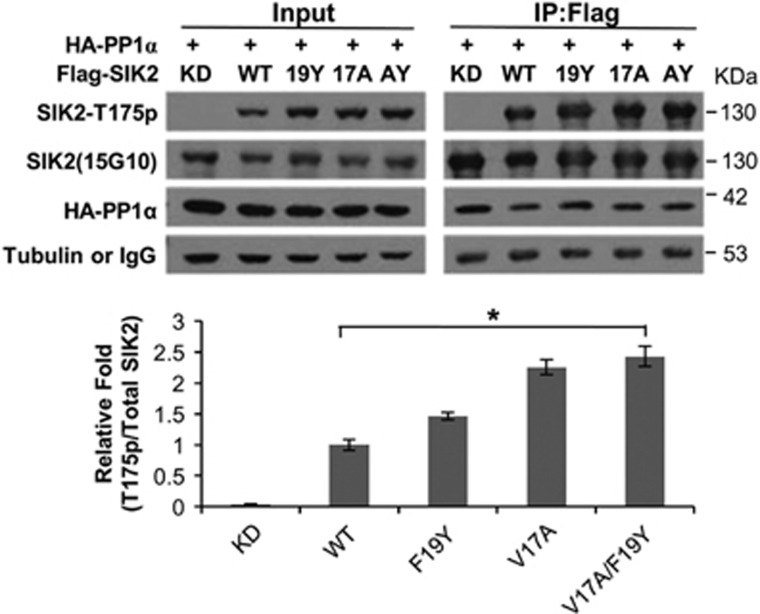
The kinase activity of SIK2 is negatively regulated by PP1. The 293T cells were co-transfected with HA-PP1*α*-WT, as well as Flag-SIK2-WT, -KD, -F19Y, -V17 A or -F19Y/V17A. The cell lysates were immunoprecipitated with anti-Flag agarose. The histogram shows relative folds of quantification of western blot images (*n*=2). Left panel: input control; right panel: anti-Flag immunoprecipitates; 19Y: F19Y mutant; 17A: V17A mutant; AY: V17A/F19Y double mutant

Negative regulations of SIK2 by PKA-mediated phosphorylation of Ser358 and CaMK1/4-mediated phosphorylation of Thr484 have been reported.^30, 34^ We have demonstrated that SIK2 and PP2A could form a stable complex and preserve both its kinase and phosphatase activities.^41^ Having addressed the mode of SIK2 activation (i.e., upstream kinase activation-targeted SIK2 followed by autophosphorylation), we would like to address how SIK2/Thr175 phosphorylation is negatively regulated. To investigate the potential protein phosphatase(s) responsible for the inactivation of SIK2 (i.e., dephosphorylation of SIK2/ Thr175), we surveyed its amino-acid sequence and noticed that a consensus PP1-binding box, 16-PVxF-19, is located in the N-terminal region. We performed co-transfection of 293T cells with Flag-tagged SIK2-WT, SIK2-KD, SIK2-V17A, SIK2-F19Y, SIK2-V17A-F19Y and HA-PP1*α*. Western blot of immunoprecipitates was probed with anti-SIK2/Thr175 phospho-specific antibody, SIK2 mAb, anti-HA and anti-tubulin antibodies. As shown in Figure 6, the phosphorylated SIK2/Thr175 levels of both SIK2-V17A and SIK2-F19Y mutants were substantially higher compared with that of SIK2-WT. However, the level of immunoprecipitated PP1*α* remains unchanged. These results suggest that the phosphorylated SIK2-Thr175 level is negatively regulated by PP1*α* in a PVxF motif-dependent manner. However, these results also suggest that PP1*α* can exist in both wild-type- and mutant SIK2-containing complexes.

## Discussion

In this study, we demonstrated that the proteasome activity was inhibited and autophagy was promoted in peritoneal mesothelial cells exposed to PD solution both *in vitro* and *in vivo*. The interaction between UPS and autophagy system, and the role of SIK2 in maintaining viability of peritoneal mesothelial cells during PD have not been previously reported with respect to peritoneum integrity.

Maintaining peritoneum membrane integrity and the viability of peritoneal mesothelial cells is crucial to long-term success of PD therapy.^7, 10^ When exposed to PD solutions, we found that the proteasome activity was inhibited in peritoneal mesothelial cells; however, the mesothelial cell death process under proteasome inhibition was decreased initially by autophagy promotion. These data suggest that the coordinated and complementary relationship between the two catabolic systems help mesothelial cells survive under PD solution exposure. To our knowledge, this is the first study identifying the survival pathways and their cross-talk in peritoneal mesothelial cells under stress induced by PD therapy. Our results indicate that PD solution, much like MG132 treatment, causes the inactivation of proteasome activity in peritoneal mesothelial cells. Under potential connection of UPS and autophagy,^26, 27^ the autophagosome formations are promoted, which maintain the viability of mesothelial cells during PD.

The mechanism by which upregulation of autophagy reduces the cell death associated with proteasome inhibition is important to the viability of mesothelial cell. In this study, we explore that SIK2 links UPS and autophagy. We found that the activated SIK2 could stimulate autophagy and promote the viability of mesothelial cells during PD. Although the high osmolarity and bioincompatibility of PD solutions may cause ER stress. However, ER stress cannot be reflected by the significant elevation of conventional ER stress indicators. This could be the result of elevation of ER stress markers by chronic treatment of PD. Rather, we noticed that the SIK2 protein level was significantly increased in the non-ionic detergent buffer-extractable fraction. In this paper, we have also uncovered that SIK2 can be extracted upon M*β*C or PD solution treatment and is likely located in the cholesterol-rich (i.e., lipid raft) fraction of ER membrane.^42, 43^ This observation raised an intriguing finding that PD solution is actually an effective proteasome inhibitor and it works through this mechanism-activating SIK2 indirectly. The present data are the first report to indicate the important role of SIK2 in mesothelial cells. Our results clearly established that the activation of SIK2 by inhibition of proteasome is crucial to the survival of mesothelial cells. Manipulating SIK2 expression could modulate the activity of autophagy and affect the survival of mesothelial cells.

Conventional PD solution uses glucose as an osmotic agent to assist ultrafiltration. In all, 7.5% icodextrin, a starch-derived glucose polymer, is a newer generation of PD solution being used as an alternative in PD patients with ultrafiltration failure.^44^ A major advantage of the 7.5% icodextrin is that a negative glucose balance can be achieved during dialysis.^45^ Whether 7.5% icodextrin may also work through disturbing lipid raft on the plasma membrane in addition to the inhibition of proteasome is an interesting question. The osmolarity of PD solution containing 7.5% icodextrin is 284 mOsmol/l, while that of 4.25% dextrose is 485 mOsmol/l. Although the osmolarity is significantly different, our present results have clearly shown that SIK2 can be activated by either 4.25% dextrose or 7.5% icodextrin, and has an important role in protecting mesothelial cells. These results suggest that osmolarity of PD solution may not be the major factor contributing to proteasome inhibition and SIK2 activation in mesothelial cells. Combined with our current results, additional studies focusing on the components of PD solutions involved in SIK2 activation and autophagy promotion will enhance our understanding of improving the biocompatibility of PD solution.

To manipulate SIK2 expression, we need to understand the mode of activation of SIK2 as well as the associated negative regulations. Most functional studies of the SIK2 use genetic knockdown strategy. More physiological and biochemical studies are needed to elucidate the *in vivo* functions of SIK2. To achieve this goal, we undertook investigations on the mode of SIK2 activation, stability regulation and characteristics in non-cancerous somatic cells. Inhibition by acetylation of Lys residues in the ATP-binding pocket and reactivation by HDAC6 represent a physiologically relevant regulation. Another physiologically significant regulation is inhibition of proteasome as demonstrated by the results of this study and the previous publications.^26, 39^ Finally, SIK2 has a PVXF box for PP1. Our results showed that phosphorylated SIK2/Thr175 could be dephosphorylated and inactivated by PP1. Additionally, SIK2 and PP2A complex possesses both kinase and phosphatase activities.^41^ Our study indicated that SIK2 activity possesses relevant physiological functions and is controlled by multilayer regulations.

The function of SIK2 in cell survival has been clearly demonstrated in the previous and the present studies.^26, 39^ SIK2-depleted cells fail to complete the cell cycle. Our previous studies indicated that SIK2 cooperates with VCP/p97 in regulating ERAD,^39^ has important roles in autophagy when proteasome function is compromised and interacts with PP2A in regulating CaMKI (Ca2(+)/calmodulin-dependent protein kinase I) activity.^41^ Notably, when C-terminal of UBA-containing portion was truncated, SIK2 became constitutively activated. SIK2-KD mutant (by either K49M or K53Q mutation) was deficient in ATP binding and also failed to be phosphorylated at Thr175.^26^ Together, these results suggest that SIK2 is phosphorylated and activated by upstream kinase(s) at regions outside the kinase domain. When this happens, SIK2 becomes activated and Thr175 is autophosphorylated. Based on the published and our present results, the candidate upstream kinases responsible for the activation of SIK2 could be LKB1 and/or JNK. HeLa cells are deficient in LKB1, yet SIK2 can be activated by MG132-mediated inhibition of proteasome. This result suggests that JNK may be one of the kinases that lead to SIK2 activation in HeLa cells. Together, these results suggest that SIK2 activation is likely via a two-step mechanism, upstream kinase(s) relieving the autoinhibitory conformation of SIK2 molecule followed by the autophosphorylation of Thr175 and the activation of kinase activity. Further study to delineate the regulatory mechanism of SIK2 activation will provide new insights into the viability of mesothelial cells and, furthermore, achieve targeted modulation of peritoneal membrane integrity.

In conclusion, the present study found that SIK2 has an important role for promoting the viability of mesothelial cells during PD therapy. Proteasome inhibition of mesothelial cells is observed during PD therapy; this mobilizes and activates SIK2 to promote autophagy and protect mesothelial cells from apoptosis. Maintaining or boosting the activity of SIK2 may promote the viability of peritoneal mesothelial cells and protect mesothelial cells against stress induced by PD solution exposure during PD. These findings enhance our understanding of the mechanism of peritoneal mesothelial cell survival and facilitate the development of new therapy strategies, such as maintaining or restoring the integrity and function of peritoneal membrane, leading to the long-term success of PD treatment.

## Materials and Methods

### Plasmids, constructs and antibodies

The pBluescript II vector encoding SIK2 sequence (KIAA0781) was from HUGE protein database (Kazusa DNA Research Institute, Kisarazu, Chiba, Japan). The DNA fragment encoding SIK2 was excised using *Sal*I and *Xho*I sites in the pBluescript-SIK2 vector and in turn subcloned into pCMV-tag mammalian expression vector (Stratagene, Santa Clara, CA, USA). Flag-SIK2-KD (kinase dead, K49M) was detailed elsewhere.^26, 41^ pLKO.1-shRNA: shLuc and shSIK2 were obtained from National RNAi Core Facility (Institute of Molecular Biology/Genome Research Center, Academia Sinica, Taipei, Taiwan). The rabbit antibodies against PARP-1 and LC3 and monoclonal antibodies against PP2Ac and PP2Aa were purchased from Cell Signaling (Danvers, MA, USA). The mouse monoclonal antibody against HA-tag was purchased from Sigma (St. Louis, MO, USA). Monoclonal antibodies to SIK2 (clone 15G10), anti-tubulin-*α* (clone 10D8) and rabbit anti-SIK2/T175p and anti-Flag antibodies were generated in our lab. Okadaic acid was purchased from Sigma. PP1*α* was cloned by PCR from 293T cells. Flag-tagged PP1*α* was obtained by cloning the *Bam*HI/*Eco*RV fragment into pCMV-tag2B, while HA-tagged PP1*α* was derived from cloning *Bam*HI/*Eco*RI fragment into pcDNA3/HA, respectively. The following primers were used in PCR: PP1*α* -*Bam*HI-F′, 5′-AAAGGATCCGCCATGTCCGACAGCGAGAA-3′ PP1*α*-*Eco*RV-R′: 5′-GGGGATATCGGGCTATTTCTTGGCTTTGGC-3′ and PP1*α* -*Eco*RI-R′: 5′-GGGGAATTCGGGCTATTTCTTGGCTTTGGC-3′:

### Generation of SIK2 and PP1*α* mutants

pCMV-Flag-SIK2/T175A, pCMV-Flag-SIK2/F19Y, pCMV-Flag-SIK2/V17A, pCMV-Flag-SIK2/V17A/F19Y and pcDNA3-HA-PP1a/C291R mutants were generated by using the following primers and the QuikChange Site-Directed Mutagenesis Kit (Stratagene, Santa Clara, CA, USA) according to the manufacturer's instructions: SIK2-F19Y, 5′-GGTCCGGGTGGGGTACTACGACATCGAGG-3′ SIK2-V17A, 5′-CCGGTCCGGGCGGGGTTCTACGACATC-3′ SIK2-V17A/F19Y, 5′-CCGGTCCGGGCGGGGTACTACGACATCGAG-3′ PP1*α*-C291R, 5′-GTGGACGAGACCCTCATGAGATCTTTCCAGATCC3′.

### Cell cultures and transfections

Human peritoneal mesothelial cells from effluent were obtained by centrifugation of dialysis fluid taken from patients undergoing nocturnal exchanges with dialysis solutions. Patients used 1.5- or 2-L PD bags with four daily exchanges. Glucose concentration of PD solution with 2.5% dextrose or 7.5% icodextrin in nocturnal exchange depends on patient's clinical condition and peritoneal membrane transport characteristics. The mesothelial cells were separated by centrifugation at 400 × *g* at 4°C for 10 min. Cells were cultured in Earle's M199 medium, 20% fetal calf serum, 50 U of penicillin per ml, 50 *μ*g of streptomycin per ml and 2% Biogro-2 (containing insulin, transferrin, ethanolamine and putrescine) (Biological Industries, Cromwell, CT, USA). The identity of mesothelial cells was confirmed by their uniform cobblestone appearance at confluence, the presence of surface microvilli, the absence of factor VIII-related antigen staining and the presence of cytokeratin and vimentin.

Cells were seeded on 6-well plates, cultured to 80% confluence and then changed to fresh culture medium (including 20% FBS) incubating for 1 h. After 1 h incubation, the medium is discarded and cells were treated with fresh culture medium, 2.5% dextrose (Dianeal PD-2 solution with 2.5% dextrose; Baxter Healthcare Corporation, Deerfield, IL, USA), 4.25% dextrose (Dianeal PD-2 solution with 4.25% dextrose; Baxter Healthcare Corporation) and 7.5% icodextrin (Extraneal PD solution with 7.5% icodextrin; Baxter Healthcare Corporation) PD solution for 2 h. A total of 25 and 5 *μ*M MG132 (Biomol, Farmingdale, NY, USA) were used for 2 and 16 h, respectively.

Human newborn foreskin fibroblasts Hs68 cells were cultured in Dulbecco's modified Eagle's medium (DMEM) containing 10% fetal bovine serum (FBS) at 37 °C. Human peritoneal mesothelial LP9 cells (NIA Aging Cell Culture Repository Catalog ID: AG07090) were cultured in a medium containing 1:1 mixture of M199 (Gibco; 11150-059, Waltham, MA, USA) and F12 (Gibco; 11765 replaced MCDB 110) supplemented with 20% FBS (HyClone, Logan, Utah, USA; SH30071), 10 ng/ml EGF (R&D; 236-EG, Minneapolis, MN, USA), 0.05 *μ*g/ml hydrocortisone (Sigma; H6909) and 50 U/ml antibiotics (Corning, Corning, NY, USA; BII03-033-1B). The cultures were subcultured every 3–4 days in a 1:3 split. LP9 cells were scrapped off from the Petri dish (6 × 10 cm) and washed with phosphate-buffered saline (PBS). A measure of 0.4 ml of 10 mM Tris-HCl (pH 7.4) was added to the above cells, which were then disrupted using a dounce homogenizer. The lysate was immediately adjusted to 0.25 M sucrose, 1 mM MgCl_2_ and centrifuged at 1000 × *g* for 10 min at 4 °C to remove nuclei and cell debris. The 293T cells were cultured in DMEM supplemented with 10% FBS and 100 U/ml penicillin–streptomycin (Gibco) at 37 °C in a humidified incubator containing 5% CO_2_.

The 293T cells were transfected using the calcium phosphate precipitation method. At 12–16 h after transfection, the culture medium was refreshed for recovery at ~8 h and treated with different drugs.

### Lentivirus-shSIK2-mediated knockdown of SIK2

At 16 h before lentivirus-shSIK2 infection, 0.5 × 10^5^ of HEK293T cells were plated in a well of 6-well plate in 2 ml medium. Lentivirus-shSIK2 in DMEM was added to the culture cells at an MOI of 20 and incubated for 48 h.

### Preparation of WCEs

The cells were washed with PBS (pH 7.4) and pelleted by spinning for 3 min at 3000 r.p.m. Cell pellet was lysed directly with cold whole-cell extraction (WCE) buffer (0.15 M NaCl, 20 mM Tris (pH 7.6), 0.2% Triton X-100, 1 mM PMSF, 1 *μ*g/ml leupeptin, 1 *μ*g/ml pepstatin A, NaF, Van, sodium butyrate and 1 mM DTT, 25 mM NEM). Lysis was achieved by performing pipetting. The cell lysates were kept on ice for 20 min, and then centrifuged for 15 min at 14 000 r.p.m. at 4 °C. The pellet was dissolved in 1 × sample buffer and used for further analysis.

### Immunoblot analysis

Proteins in the lysates were separated by SDS-polyacrylamide gel electrophoresis and transferred to Hybond-C membrane (Amersham Bioscience, Piscataway, NJ, USA). The membrane was blocked with 5% non-fat milk in PBST at room temperature for 30 min and then probed with primary antibody at room temperature for 1 h. After having been washed with PBST three times, the membrane was probed with HRP-conjugated secondary antibody at room temperature for 1 h and washed with PBST three times. The membrane was incubated with HRP-ECL (Perkin-Elmer, Waltham, MA, USA) and exposed to X-ray film.

### Immunopreciptation assay

WCEs were incubated with anti-Flag M2 beads (Sigma). After being gently rotated for 1.5 h at 4 °C, the beads were washed with WCE buffer three times. The precipitated Flag-tagged proteins were eluted by 1 × SDS-PAGE sample buffer (8 M urea, 300 mM Tris-HCl (pH 6.8), 20% *β*-mercaptoethanol, 9.2% SDS and 0.2% bromophenol blue).

### Immunofluorescent staining

Cells in coverslips were washed with PBS, fixed with 2% (v/v) formaldehyde for 30 min, washed with cold PBS for three times and permeablized with PBS containing 0.5% (v/v) Triton X-100 for 5 min. Cells were blocked with 1% BSA in PBS for 30 min and incubated with indicated antibodies diluted in 1% BSA/PBS. Alexa 594-conjugated goat anti-mouse IgG (Molecular Probes Inc., Waltham, MA, USA) and Alexa 488-conjugated goat anti-rabbit IgG were used as secondary immunofluorescent dyes. Hoechst was used to visualize DNA. Stained cells were analyzed with Zeiss fluorescence microscopy (ZEISS, Oberkochen, Germany).

### Flow cytometry

Cells were washed two times with cold PBS and then resuspended in Annexin V-binding buffer (10 mM HEPES, pH 7.4, 140 mM NaCl, 2.5 mM CaCl_2_) with a concentration of 1x10^6^ cells per ml. One hundred microliters of cell suspension was transferred to a 5 ml test tube and 5 *μ*l of FITC Annexin V was added. The cells were gently vortexed and incubated for 15 min at 25 °C in the dark. After 400 *μ*l of Aannexin V-binding buffer was added to each tube, and the cells were analyzed by flow cytometry.

### Proteasome activity

Cells were washed with PBS and incubated with 7.5% icodextrin or 25 mM MG132 for 2 h. WCEs were prepared. An aliquot was incubated with *N*-succinyl-Leu-Leu-Val-Tyr-7-amido-4-methylcoumarin (Sigma-Aldrich, St. Louis, MO, USA). When a C-terminal 7-amino-4-methylcoumarin-containing peptide was hydrolyzed, the fluorescent compound 7-amino-4-methylcoumarin was released. This compound was monitored by excitation at 345 nm and emission at 445 nm in 45 mM Tris-HCl buffer, pH 8.0, containing 5 mM DTT and 10 mM CaCl_2_ incubated for 1 h at 37 °C in a 96-well plate.

### Statistical analysis

All data are presented as means±S.D. The differences between groups were analyzed by the Mann–Whitney *U*-test. *P*<0.05 was considered to be significant.

## Abbreviations

AMPK: AMP-activated protein kinase
CaMKI: Ca2(+)/calmodulin-dependent protein kinase I
ER: endoplasmic reticulum
ERAD: ER-associated degradation
HDAC6: histone deacetylase 6
LC3: microtubule-associated protein 1A/1B-light chain 3
NBR1: neighbor of BRCA1 gene 1
PD: peritoneal dialysis
PP2A: protein phosphatase 2A
SIK: salt-inducible kinase 2
UPS: ubiquitin-proteasome system.
